# Yerba Maté and its impact on glycemic control and metabolic health: a systematic review and meta-analysis

**DOI:** 10.3389/fendo.2025.1641592

**Published:** 2025-10-30

**Authors:** Daiping Li, Liantian Yue, Xuchao Peng, Ling Chen, Taiping Lin, Li Huang, Yadong Liu, Jirong Yue, Xiaoli Huang

**Affiliations:** ^1^ Department of Geriatrics and National Clinical Research Center for Geriatrics, West China Hospital of Sichuan University, Chengdu, Sichuan, China; ^2^ School of Sport, Exercise and Health Sciences, Loughborough University, Loughborough, United Kingdom

**Keywords:** Yerba Maté (Ilex paraguariensis), glycemic control, systematic review, meta-analysis, randomized controlled trials (RCT)

## Abstract

**Purpose:**

Yerba Maté, a traditional South American herbal infusion abundant in bioactive compounds, has been suggested to offer health benefits including lipid regulation and weight management. However, existing evidence remains inconclusive. This systematic review and meta-analysis seeks to conduct a comprehensive evaluation of the effects of Yerba Maté consumption on metabolic health outcomes using data from randomized controlled trials (RCTs).

**Methods:**

In accordance with the PRISMA guidelines, a comprehensive systematic review and meta-analysis of RCTs was conducted, encompassing studies published up to January 2025. Studies were systematically retrieved from MEDLINE, EMBASE and The Cochrane Central Register of Controlled Trials without any language restrictions. The review included RCTs that evaluated the impact of Yerba Maté on metabolic health indicators. Meta-analyses were performed using Review Manager (RevMan 5.4) when two or more studies from the same comparator provided sufficient data. Quality assessment were assessed using the revised Cochrane risk-of-bias tool for randomized trials (RoB 2) tool. The overall quality of evidence was evaluated using the GRADE (Grading of Recommendations, Assessment, Development and Evaluations) method.

**Results:**

A total of 1294 studies were initially identified, of which 13 RCTs met the inclusion criteria. The study population includes dyslipidemic volunteers, overweight and obese and non-dyslipidemic, normal-weight volunteers. The results with pre-diabetes patients suggest significant decreases in postprandial glucose (MD -12.76, 95% CI -16.78, -8.74; N = 2), HbA1c (MD -0.37, 95% CI -0.56, -0.18; N = 2), and the homeostatic model assessment index (HOMA index) (MD -0.24, 95% CI -0.37, -0.11; N = 2), though further research is needed to confirm these findings. No significant effects were found on triglycerides, total cholesterol, HDL-C, LDL-C, fasting glucose, fasting insulin, waist circumference, or BMI. Adverse events included mucosal irritation, insomnia, tachycardia, angina, headache, and gastrointestinal discomfort.

**Conclusion:**

Yerba Maté consumption may demonstrate favorable effects on glycemic control, though its impact on lipid profiles and weight management appears to be limited.

**Systematic Review Registration:**

https://www.crd.york.ac.uk/PROSPERO/, identifier CRD42023369270.

## Introduction

Ilex paraguariensis, or Yerba Maté, is a South American plant mainly grown in northern Argentina, Paraguay, Uruguay, and the south of Brazil (1). The history of this plant is long, and its cultural roots are deep in South America, where it is extensively used in social activities and day-to-day consumption. Because of its unique bitter taste and pleasant aroma, it’s a beverage popular not only in South America but also increasingly globally. This beverage constitutes a sophisticated matrix comprising bioactive constituents, including chlorogenic acid, polyphenolic compounds, saponins, and methylxanthine derivatives such as caffeine, theobromine, and theophylline. The predominant bioactive fraction consists of chlorogenic acids, which account for 71–76% of total polyphenols, together with methylxanthines, saponins and flavonoids (2, 3). Of them, chlorogenic acid is noteworthy as a lipid regulator and lipid oxidizing reduction that may be beneficial in preventing cardiovascular disease (4–6). Polyphenols combat oxidative stress and inflammation by neutralizing reactive oxygens species (7, 8); saponins may influence lipid metabolism by modulating intestinal lipid absorption (9, 10).

Epidemiological and clinical studies indicate Yerba Maté may influence lipid homeostasis, decreasing total cholesterol, LDL-C (low-density lipoprotein cholesterol) and triglycerides as well as increasing HDL-C (high-density lipoprotein cholesterol) (11–14). The behavior of signaling pathways may mediate these effects, e.g., the AMPKα-LXRα/SREBP-1c axis controlling lipolysis in adipose tissue (15, 16). According to the IDF (International Diabetes Federation) Diabetes Atlas 2025, in 2024, there were 589 million adults aged 20–79 living with diabetes globally, with a prevalence rate of approximately 11.1%. Globally diabetes and its complications caused over 3.4 million deaths, accounting for 9.3% of total global deaths. Although there is preliminary evidence that suggests Yerba Maté may play a role in modulating postprandial glycemic responses and decreasing HbA1c (glycated hemoglobin) levels (17, 18), we argue that these effects are likely due to the psychostimulant effect of the included xanthines. Furthermore, the mechanisms underlying these effects may include enhanced insulin sensitivity and the mechanism of action of glucose metabolism, which can be regulated by the PI3K-AKT signaling pathway that controls insulin resistance-associated genetic expression (19). Preliminary clinical investigations suggest that Yerba Maté may exert beneficial effects on weight management through multiple mechanisms, including pancreatic lipase inhibition, modulation of gastric motility, and enhancement of energy expenditure (20, 21). Several pilot studies have demonstrated modest yet statistically significant body weight and waist-to-hip ratio decreases in the supplemented group vs. placebo cohorts (20).

Existing studies also indicate that consumption of Yerba Maté may offer potential health benefits, but the results of these studies are inconsistent. As such, the aim of this systematic review is to provide a comprehensive review of the effects of Yerba Maté about health outcomes, such as lipid profiles, blood glucose levels, weight management and potentially harmful effects of consumption, by searching for evidence published in randomized controlled trials (RCTs).

## Methods

This systematic review was conducted in strict adherence to the PRISMA (Preferred Reporting Items for Systematic Reviews and Meta-Analyses) guidelines. The protocol was registered on the International Prospective Register of Systematic Reviews (PROSPERO), with the registration number CRD42023369270.

### Search strategy

We searched all publications from database inception to 15 January 2025 using three electronic databases: EMBASE, MEDLINE, and The Cochrane Central Register of Controlled Trials. No language restrictions were applied. The search strategy included the following terms: (exp ilex paraguariensis/or ((“mate” or “chimarrao” or “ilex paraguariensis” or “terere” or “yerba-mate”).ab.)) AND (randomized controlled trial.pt. or controlled clinical trial.pt. or randomized.ab. or placebo.ab. or drug therapy.fs. or randmo*.ab. or trial.ab. or groups.ab. not exp animals/not humans.sh.). To optimize the search outcomes, suitable term combinations and truncations were carefully chosen and customized for each database. The detailed search strategies are comprehensively outlined in **Supplementary Table 1**. To ensure the identification of all potentially relevant studies, reference lists of included publications were manually scrutinized. Other study sources are mainly supplemented through reference list searching.

### Eligibility criteria

The present review included RCTs that assessed the effects of Yerba Maté intake on metabolic health indicators. The inclusion criteria for the studies are systematically presented in **Table 1** according to the PICOS principle. No restrictions were placed on language or time. Studies were excluded based on the following criteria: (1) Non-original research publications including reviews, letters, posters, conference abstracts, case reports and opinion pieces; (2) Studies where Yerba Maté was combined with other plants or supplements; (3) Studies without available information of interest.

**Table 1:** PICOS criteria for inclusion of studies.

Parameter	Criteria
Condition or domain being studied	dyslipidemia, T2DM/pre-diabetes, lFG or lGT (pre-diabetes status), at most one criteria of metabolic syndrome, obese. consumption of Yerba Maté as a possible lipid-lowering/glucose-reducing/anti-obesity compound
Patient/population	Individuals of all ages and genders
Intervention	Any form of Yerba Maté consumption (tea, extract, tablets, or capsules)
Comparison	Placebo, water, or other dietary interventions
Outcomes	Serum levels of triglycerides, total cholesterol, high-density lipoprotein cholesterol (HDL-C), low-density lipoprotein cholesterol (LDL-C), fasting glucose, fasting insulin, postprandial glucose, glycated hemoglobin (HbA1c), homeostatic model assessment index (HOMA index), waist circumference, body mass index (BMI), and adverse events (AEs)
Study design	Randomized controlled trials (parallel and crossover designs)

### Data extraction

Two investigators (Li Huang and Yadong Liu) independently screened titles and abstracts, followed by full-text reviews of eligible studies. Data were extracted according to predefined criteria. Disagreements were resolved through consensus discussions involving a third independent reviewer (Jirong Yue). The extracted data included: first author’s last name, publication year, country, study design, sample size (intervention and control groups), mean age, gender, dosage of Yerba Maté, study duration, treatment duration, and baseline and outcome data for triglycerides, total cholesterol, HDL-C, LDL-C, fasting glucose, fasting insulin, postprandial glucose, HbA1c, waist circumference, body mass index (BMI), homeostasis model assessment (HOMA) index, and adverse events (AEs).

### Quality assessment

Two investigators (Daiping Li and Liantian Yue) independently assessed the risk of bias in the included RCTs using the RoB 2 tool. The risk of bias in parallel RCTs was assessed using the RoB 2.0 tool for RCTs (Version dated 22 August 2019). For crossover trials, the RoB 2.0 tool specific to crossover studies was utilized (Version dated 18 March 2021). Both tools evaluate the risk of bias across five domains: randomization process, deviations from intended interventions, missing outcome data, measurement of the outcome, and selection of reported results. Disagreements were resolved through discussion with a third author (Jirong Yue). In cases where pertinent information was lacking, we proactively contacted the respective authors for clarification.

The overall quality of the evidence was evaluated using the GRADE (Grades of Recommendation, Assessment, Development and Evaluation) approach (21–23). The GRADE framework assesses the quality of evidence across five domains: risk of bias, directness of evidence, heterogeneity, precision of effect estimates, and risk of publication bias. The quality of evidence was independently evaluated by two reviewers (Daiping Li and Liantian Yue). In cases of discrepancies, an additional experienced rater was consulted (Jirong Yue).

### Statistical analysis

The primary outcomes assessed in this study included triglycerides, total cholesterol, HDL-C, LDL-C, fasting glucose, fasting insulin, HbA1c, postprandial glucose, waist circumference, BMI, and AEs. All outcomes were reported as the change from baseline values. The incidence of adverse events was also analyzed.

Meta-analyses were performed using Review Manager (RevMan 5.4) when two or more studies from the same comparator provided sufficient data (i.e., mean difference [MD], standard deviation [SD], and number of participants in each intervention group). The I² statistic was calculated to quantify between-trial heterogeneity and inconsistency. When summary measures were not reported as MD and SD, previously published conversion tools were utilized (24). The randomized crossover trial used data from both intervention periods for analysis in the meta-analysis.

We quantified heterogeneity with I², interpreting 0-40% as low, 40-60% as moderate, and 60-90% as high (25). All meta-analyses were conducted using a random-effects model irrespective of I² value, in accordance with Cochrane Handbook recommendations. The chi-square *P* value was calculated to assess the statistical significance of heterogeneity, with *P* ≤ 0.05 indicating significant heterogeneity among the studies included in the meta-analysis. Sensitivity analyses were performed to evaluate the robustness of the results by: (1) excluding low-quality studies and (2) excluding studies with fewer than 10 participants.

## Results

### Study selection

**Figure 1:**
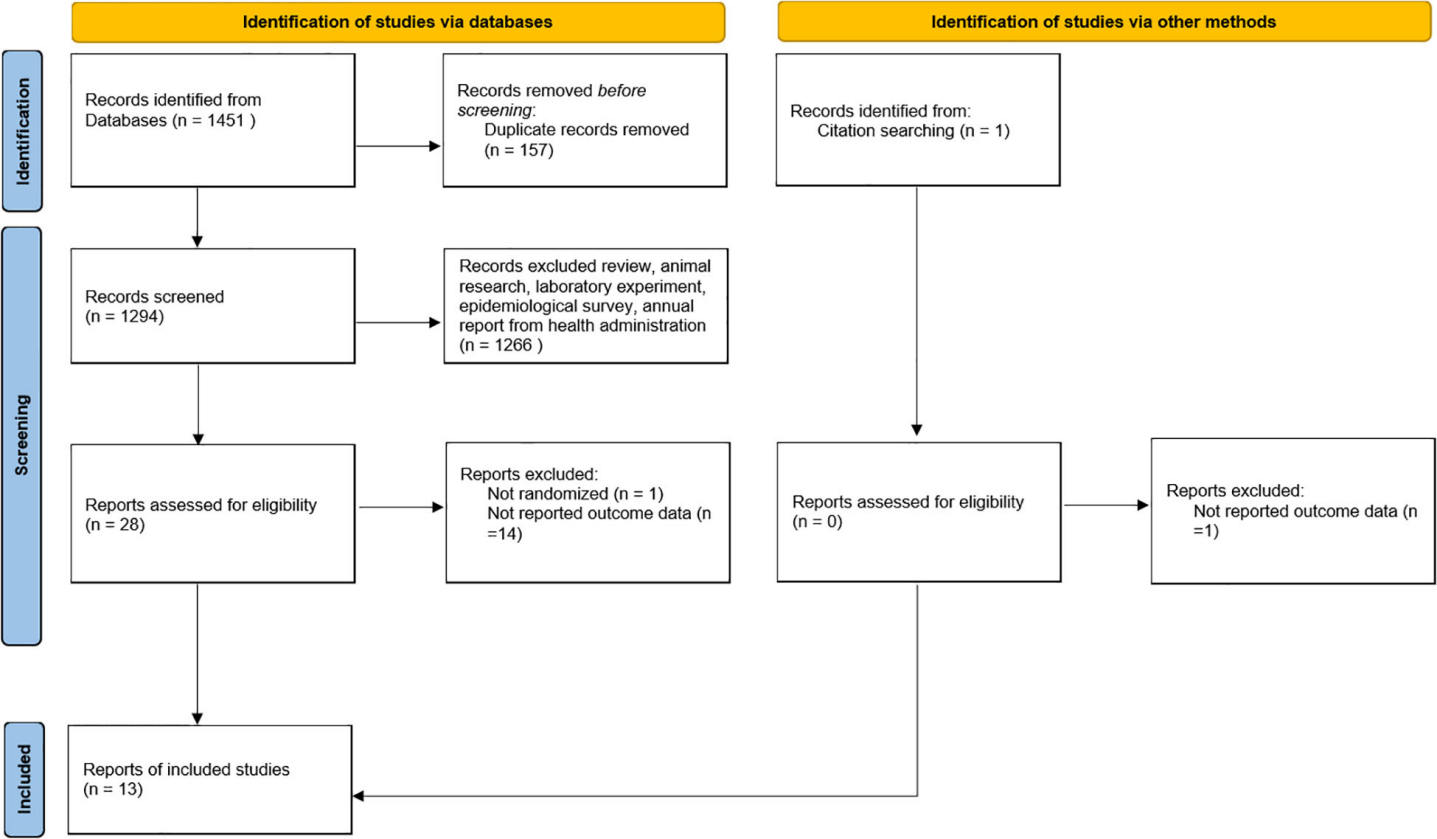
Flow diagram of identified citations and included studies.

A total of 1451 citations were identified through the initial database search. After eliminating duplicates and screening titles, 1294 citations were considered potentially relevant. During the title/abstract screening phase, 1265 studies were excluded due to failure to meet the study objectives. Full-text screening further excluded 16 studies (12, 26–40). The reasons for excluding these studies are detailed in **Supplementary Table 2**. Ultimately, 13 RCTs (17, 21, 41–51) fulfilled the inclusion criteria for this systematic review and meta-analysis. **Figure 1** is a flow diagram of the study selection process.

### Study characteristics

A detailed overview of the study characteristics is presented in **Table 2**. The 13 studies enrolled participants with ages ranging from 28 to 57 years. Among these studies, 9 were parallel RCTs and 4 were crossover RCTs. The studies were conducted across various countries: five in Brazil (17, 43, 46, 50, 51), three in Korea (21, 47, 48), two in Italy (44, 45), one in Germany (49), one in Norway (41), and one in Argentina (42). The study sample comprised healthy adults (41, 49), overweight or obese individuals (21, 42, 47, 48), participants with metabolic abnormalities (17, 43–46), and people with HIV (50, 51). Interventions were delivered as Yerba Maté capsules (21, 41, 44–51) or as Yerba Maté tea (42, 43, 49). Daily capsule doses ranged from 0.5 g (44), 1.0 g (45), 2.25 g (46), 3.0 g (21, 47, 48, 50, 51) to 5 g (41); tea doses were 20 g (17, 43) or 100 g (42). The extract used by Opala 2006 (49) (containing asparagus, green tea, black tea, guarana, maté, and kidney beans) did not specify individual amounts. Treatment durations spanned 5 days (41), 15 days (50, 51), 4 weeks (46), 6 weeks (47, 48), 60 days (17), 3 months (44, 45), 12 weeks (21, 42, 49) and 90 days (43).

**Table 2:** Characteristics of studies included in the meta-analysis.

First author and year	Location	Study design	Number of patients	Sample size I/C	Type of patients	Mean age	Gender (M/F)	Intervention group	Control group	Study duration	Dosage/day	Outcomes of interest
Areta,2018 (41)	Norway	Crossover RCT	9	9/9	Well-trained male cyclists	30	9/0	Yerba Maté capsules	Placebo	5 days	5 g	Glucose, Lactate, Free fatty acids, Glycerol, Caffeine, Paraxanthine, Adrenaline
Avena,2019 (42)	Argentina	Parallel RCT	119	not reported	Overweight (25.0 kg/m^2^ ≤BMI <32.5 kg/m^2^)	36	0/119	Yerba Maté tea	Water and dietary intervention	12 weeks	100g	Total cholesterol, Triglycerides, HDL-c, LDL-c, Waist circumference, Body weight, BMI, Body fat, BFM
Boaventura,2012 (43)	Brazil	Parallel RCT	51	25/26	Dyslipidemic volunteers	47	9/42	Yerba Maté tea	Dietary intervention	90 days	20g	Total cholesterol, LDL-c, HDL-c, non-HDL-c, Triglycerides, Ferric reducing antioxidant potential, Uric acid, Reduced glutathione, Lipid hydroperoxide, Protein carbonyl, Enzyme paraoxonase-1
Derosa,2019 (44)	Italy	Parallel RCT	130	66/64	Patients with pre-diabetes	53	63/67	Glicoset 500 (Yerba Maté tablet)	Placebo	3 months	0.5g	Total cholesterol, Triglycerides, HDL-c, LDL-c, Body weight, BMI, Waist circumference, Hip circumference, Abdominal circumference, Fasting plasma glucose, Postprandial glucose, HbA_1c_, Fasting plasma insulin, HOMA index, Hs-CRP, AST, ALT, Glycemia, Impaired fasting glucose, Impaired glucose tolerance
Derosa,2021 (45)	Italy	Parallel RCT	148	72/76	Patients with pre-diabetes	54	73/75	Glicoset 1000 (Yerba Maté tablet)	Placebo	3 months	1g	Total cholesterol, Triglycerides, HDL-c, LDL-c, Body weight, BMI, Waist circumference, Hip circumference, Abdominal circumference, Fasting plasma glucose, Postprandial glucose, HbA_1c_, Fasting plasma insulin, HOMA index, Hs-CRP, AST, ALT, Glycemia, Impaired fasting glucose, Impaired glucose tolerance
Gebara,2021 (46)	Brazil	Crossover RCT	34	34/34	With at most one criteria of metabolic syndrome	50	34/0	Yerba Maté capsules	Placebo (starch)	4 weeks	2.25g (Maté extract)	Total cholesterol, Triglycerides, HDL-c, LDL-c, fasting glucose, SBP, DBP, Pulse, Waist circumference, Body weight, BMI, C-reactive protein, Intercellular adhesion molecule 1, Vascular cell adhesion molecule 1, Interleukin-6
Jung,2016 (47)	Korea	Parallel RCT	33	17/16	Overweight (25.0 kg/m^2^ ≤BMI <30.0 kg/m^2^)	44	0/33	Yerba Maté tablet	Placebo (starch)	6 weeks	3g (Maté extract)	Total cholesterol, Fasting glucose, SBP, DBP, Waist circumference, Body weight, BMI, Hip circumference, Body fat, BFM, LBM, AST, ALT, SBP, DBP, Pulse rate, Total bilirubin, Albumin, Protein
Kim,2012 (48)	Korea	Parallel RCT	60	30/30	BMI≥ 25.0 kg/m^2^	28	0/60	Yerba Maté capsules	Placebo(starch)	6 weeks	3g (Maté extract)	Waist circumference, Body weight, BMI, Body fat, BFM, LBM, Total cholesterol, Triglycerides, HDL-c, Fasting glucose, Safety
Kim, 2015 (21)	Korea	Parallel RCT	30	15/15	Obese	43	4/26	Yerba Maté capsules	Placebo	12 weeks	3g (Maté extract)	Visceral fat, Subcutaneous fat, Visceral subcutaneous ratio, Total cholesterol, LDL-c, HDL-c, non-HDL-c, Triglycerides, Free Fatty Acid
Klein,2011 (17)	Brazil	Parallel RCT	36	9/9 10/8	T2DM/pre-diabetes	57	10/26	Yerba Maté tea	Dietary intervention	60 days	20g	Total cholesterol, LDL-c, HDL-c, non-HDL-c, Triglycerides
Opala,2006 (49)	Germany	Parallel RCT	98	47/51	Healthy volunteers	42	21/77	Yerba Maté tablets	Placebo	12 weeks	two tablets	Body weight, Waist circumference, Hip, W/H, Body fat by SKF, Total cholesterol, LDL-c, HDL-c, Triglycerides, HDL/LDL, Lipoprotein (a), Fasting Insulin, Fasting glucose
Petrilli,2016 (50)	Brazil	Crossover RCT	92	92/92	HIV	45	58/34	Yerba Maté capsules	Mate-placebo	15days	3g	Hs-CRP, HDL-c, fibrinogen, Leukocytes, Lymphocytes, Neutrophils, Monocytes
Souza,2017 (51)	Brazil	Crossover RCT	92	92/92	HIV/AIDS	45	58/34	Yerba Maté tablets	Mate-placebo	15days	3g	Total cholesterol, Triglycerides, LDL-c, HDL-c, LDL(-), Apolipoprotein B-100, Apolipoprotein A1

RCT, randomized controlled trial; I, intervention group; C, control group; HbA1c, glycated haemoglobin; HOMA index, homeostatic model assessment index; Hs-CRP, high sensitivity C-reactive protein; AST, aspartate aminotransferase; ALT, alanine aminotransferase; BFM, body fat mass; LBM, lean body mass; SBP, systolic blood pressure; DBP, diastolic blood pressure; BMI, body mass index; T2DM, type 2 diabetes mellitus.

### Risk of bias

Among the nine parallel RCTs included, potential bias was identified across several domains in **Supplementary Figure 1**. Specifically, issues with the Randomization process (D1) were noted in three studies (17, 42, 43) and concerns regarding Deviations from the intended interventions (D2) were observed in two study (17, 43). One study (17) was rated as some concerns in the Missing outcome data (D3) domain. All studies, however, demonstrated a low risk of bias in the Measurement of the outcome (D4) domain. Furthermore, three studies (17, 42, 47) exhibited some concerns in the Selection of the reported result (D5) domain. Five studies (21, 44, 45, 48, 49) exhibited low risk of bias across all domains, increasing confidence in their findings.

Among the four crossover RCTs included, potential bias was identified across several domains in **Supplementary Figure 2**. Of the four crossover RCTs assessed, three studies raised some concerns about the Randomization process (D1) (41, 46, 50), and one study showed bias issues caused by menstruation and carryover effects (DS) (41). In terms of Deviations from the intended interventions (D2), one study presented a high risk of bias (50), while two studies indicated some concerns (41, 51). For Missing outcome data (D3), two studies were rated as high risk (50, 51). The Measurement of the outcome (D4) domain generally showed a low risk across studies, suggesting a reliable assessment of outcomes. As for the Selection of the reported result (D5) domain, three studies were identified with some concerns (41, 50, 51). Three studies (41, 50, 51) were rated as having some concerns in the domain of Selection of the reported result (D5). Two studies (50, 51) were classified as high risk and other two studies (41, 46) were identified with some concerns across all domains.

### GRADE assessment

Applying the GRADE framework (**Table 3**), we rated the certainty of evidence as high, moderate, or low for all outcomes. We downgraded waist circumference evidence by one level because of marked inconsistency (I²>50%). The evidence for fasting insulin and HOMA index was downgraded by one level due to detected publication bias (more than half of the studies come from the same team). Total cholesterol, triglycerides, LDL-C, and fasting glucose were downgraded by two level for very serious limitations, combining serious inconsistency and serious imprecision (wide 95% CI), resulting in low certainty. Postprandial glucose and HbA1c were downgraded by two levels due to very serious limitations, combining serious inconsistency and detected publication bias, resulting in low certainty.

**Table 3:** GRADE’s summary of findings.

Outcomes	Participants (studies)	Risk of bias	Inconsistency	Indirectness	Imprecision	Publication bias	Overall certainty	Absolute effects 95%CI
Total cholesterol	926 (11RCTS)	not serious	serious^a^ I^2^ = 89%	not serious	serious^b^	undetected	Low ⨁⨁◯◯	MD -3.97 (-12.06 to 4.12)
Triglycerides	893 (10RCTS)	not serious	serious^a^ I^2^ = 51%	not serious	serious^b^	undetected	Low ⨁⨁◯◯	MD -6.30 (-14.02 to 1.42)
HDL-C	1077 (11RCTS)	not serious	not serious I^2^ = 31%	not serious	not serious	undetected	High ⨁⨁⨁⨁	MD 0.34 (-0.50 to 1.17)
LDL-C	833 (9RCTS)	not serious	serious^a^ I^2^ = 84%	not serious	serious^b^	undetected	Low ⨁⨁◯◯	MD -3.68 (-10.63 to 3.26)
Fasting glucose	562 (7RCTS)	not serious	serious^a^ I^2^ = 96%	not serious	serious^b^	undetected	Low ⨁⨁◯◯	MD -1.46 (-7.53 to 4.62)
Fasting insulin	383 (3RCTS)	not serious	not serious I^2^ = 0%	not serious	not serious	detected^c^	Moderate ⨁⨁⨁◯	MD -0.04 (-0.78 to 0.70)
Postprandial glucose	285 (2RCTS)	not serious	serious^a^ I^2^ = 91%	not serious	not serious	detected^c^	Low ⨁⨁◯◯	MD -12.76 (-16.78 to -8.74)
HbA1c	285 (2RCTS)	not serious	serious^a^ I^2^ = 64%	not serious	not serious	detected^c^	Low ⨁⨁◯◯	MD -0.37 (-0.56 to -0.18)
HOMA index	285 (2RCTS)	not serious	not serious I^2^ = 0%	not serious	not serious	detected^c^	Moderate ⨁⨁⨁◯	MD -0.24 (-0.37 to -0.11)
Waist circumference	654 (8RCTS)	not serious	serious^a^ I^2^ = 51%	not serious	not serious	undetected	Moderate ⨁⨁⨁◯	MD -0.48 (-1.22 to -0.26)
BMI	624 (7RCTS)	not serious	not serious I^2^ = 0%	not serious	not serious	undetected	High ⨁⨁⨁⨁	MD -0.17 (-0.36 to 0.02)

^a^Downgraded for high inconsistency (I^2^ >50%).

^b^Downgraded for high imprecision (if the 95% confidence interval was very wide).

^c^Downgraded for publication bias (if more than half of the studies come from the same team).

^d^HDL-C, high-density lipoprotein cholesterol; LDL-C, low-density lipoprotein cholesterol; HbA1c, glycated haemoglobin; HOMA index, homeostatic model assessment index; BMI, body mass index.

### Impact on lipid levels

**Figure 2:**
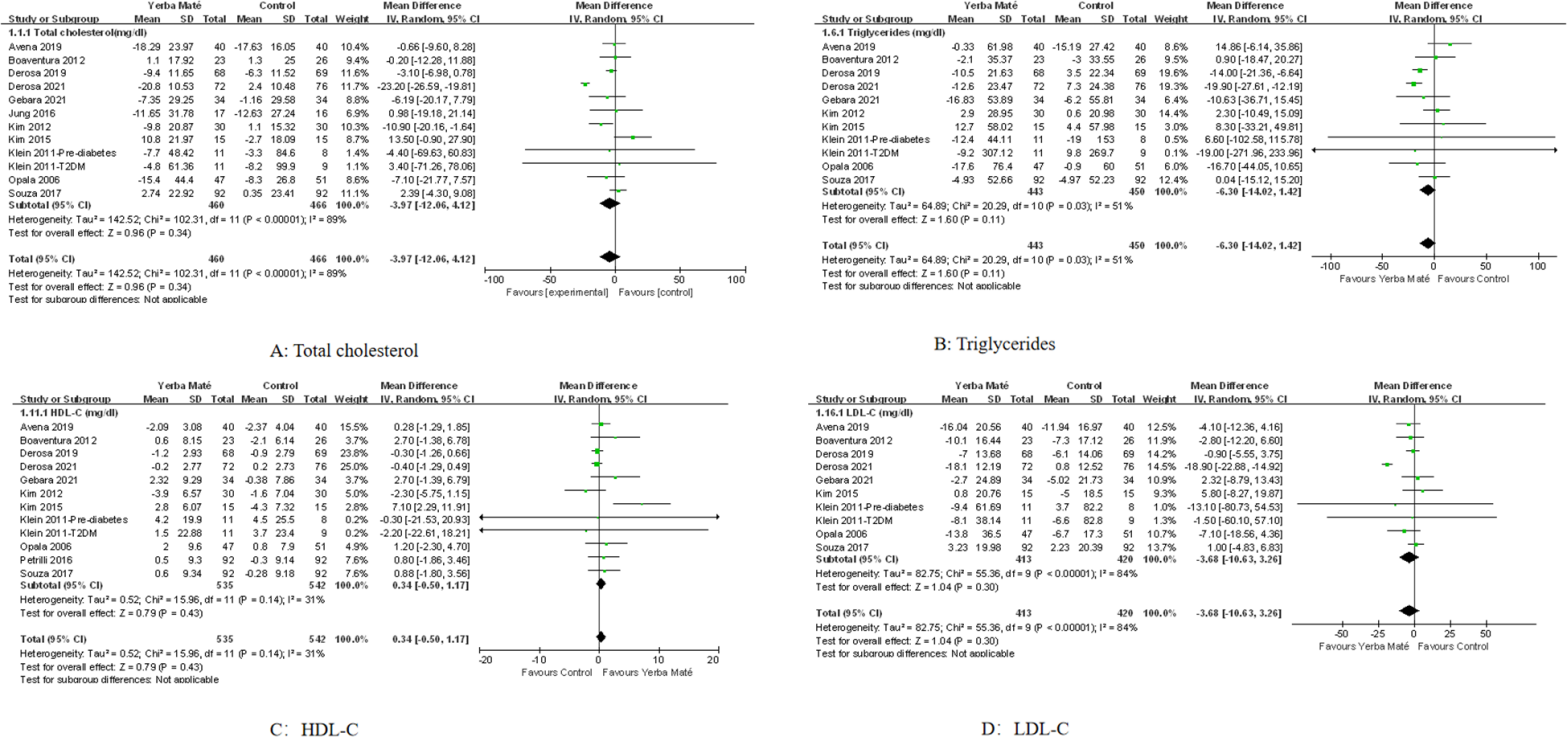
Forest plot of Yerba Maté’s effect on lipid levels. **(A)** Total cholesterol; **(B)** Triglycerides; **(C)** HDL-C (high-density lipoprotein cholesterol); **(D)** LDL-C (low-density lipoprotein cholesterol).

A total of 11 RCTs were included to assess the impact of Yerba Maté on lipid levels, with follow-up durations ranging from 15 to 90 days (**Figure 2**). Comparative analysis of Yerba Maté versus control groups demonstrated non-significant differences in total cholesterol (**Figure 2A**), triglycerides (**Figure 2B**), HDL-C (**Figure 2C**) or LDL-C (**Figure 2D**).

### Effects on blood glucose metabolism

**Figure 3:**
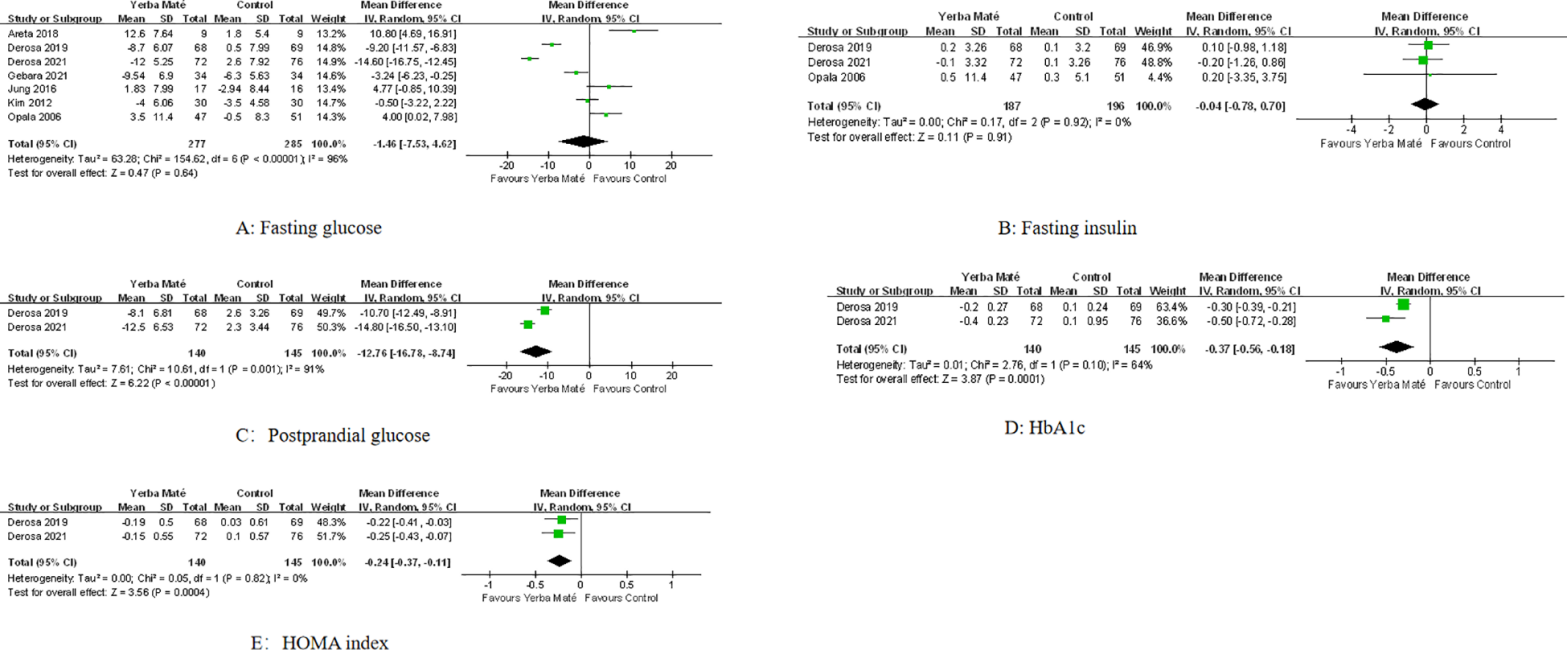
Forest plot of Yerba Maté’s effect on glycemic status. **(A)** Fasting glucose, **(B)** Fasting insulin, **(C)** Postprandial glucose, **(D)** HbA1c(glycated hemoglobin), and **(E)** HOMA index(homeostatic model assessment index).

Seven RCTs were included to evaluate the effects of Yerba Maté on glycemic status, with follow-up durations ranging from 5 days to 3 months. The quantitative analysis results are summarized in **Figure 3**. Meta-analysis revealed a significant reduction in postprandial glucose levels (MD -12.76, 95% CI -16.78 to -8.74, I² = 91%, p=0.001, N = 2) (**Figure 3C**), HbA1c levels (MD -0.37, 95% CI -0.56 to -0.18, I² = 64%, p=0.1; N = 2) (**Figure 3D**) and the HOMA index (MD -0.24, 95% CI -0.37 to -0.11, I² = 0%, p=0.82; N = 2) (**Figure 3E**). No significant differences were observed in fasting glucose levels (**Figure 3A**) or fasting insulin concentrations (**Figure 3B**) between the Yerba Maté group and the control group.

### Influence on weight management

**Figure 4:**
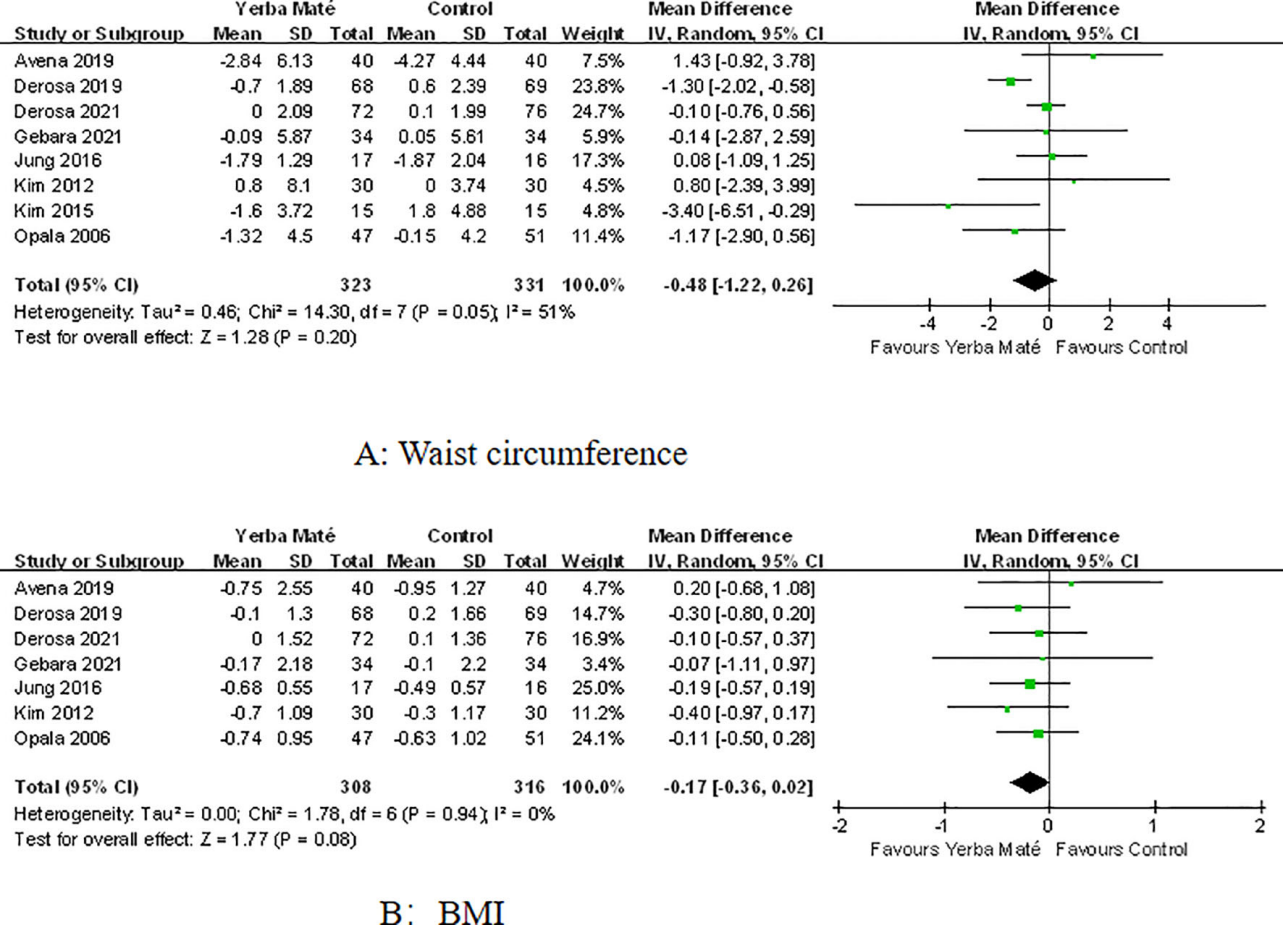
Forest plot of Yerba Maté’s effect on waist circumference and BMI. **(A)** Waist circumference, **(B)** BMI (body mass index).

Eight RCTs were included to assess the effects of Yerba Maté on waist circumference and BMI, with follow-up durations ranging from 4 weeks to 3 months (**Figure 4**). Comparative meta-analysis showed no significant differences in waist circumference (**Figure 4A**) or BMI (**Figure 4B**) between Yerba Maté and control groups.

### Subgroup analyses

**Figure 5:**
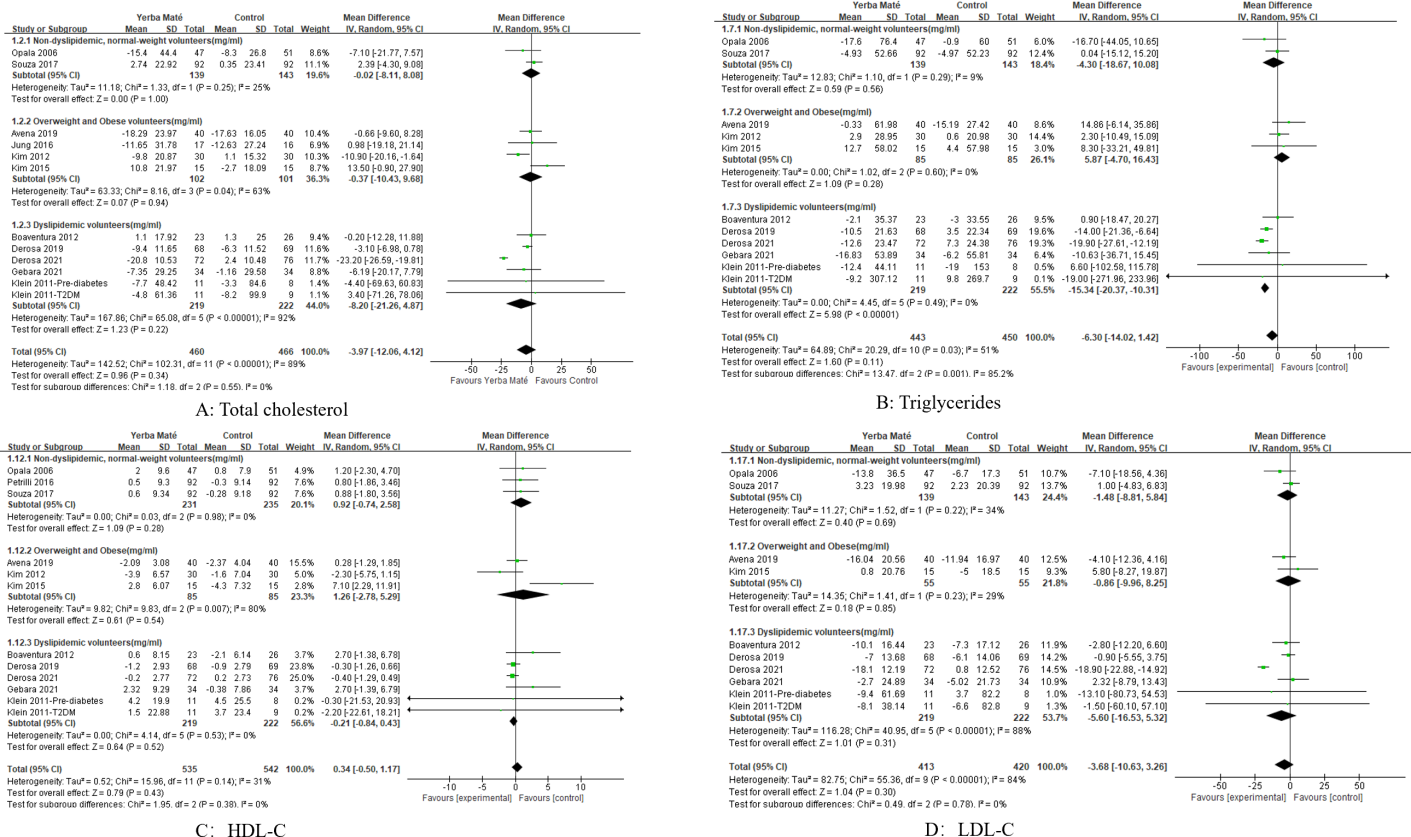
Forest plot for subgroup analysis of intervention populations on lipid levels. **(A)** Total cholesterol; **(B)** Triglycerides; **(C)** HDL-C (high-density lipoprotein cholesterol); **(D)** LDL-C (low-density lipoprotein cholesterol).

**Figure 6:**
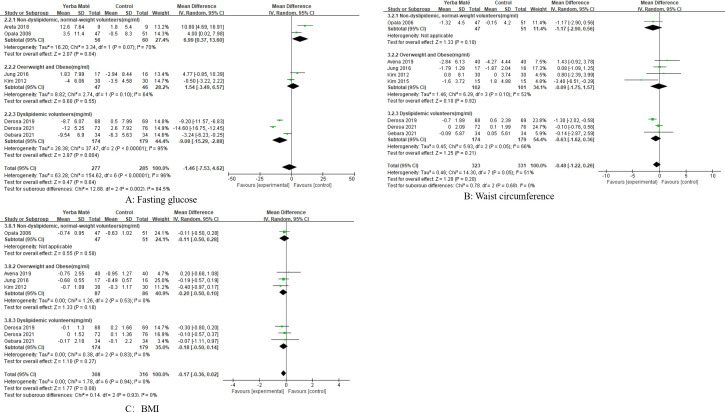
Forest plot for subgroup analysis of intervention populations on fasting glucose, waist circumference and BMI. **(A)** Fasting glucose, **(B)** Waist circumference, **(C)** BMI (body mass index).

We performed subgroup analyses of intervention population, intervention methods, and intervention duration. Subgroup analyses of intervention doses were not possible because most intervention doses were only available in 1–2 studies. We conducted a subgroup analysis of the intervention group for Yerba Maté (**Figures 5**, **6**), including: dyslipidemic volunteers, overweight and obese and non-dyslipidemic, normal-weight volunteers. Subgroup analyses found a significant difference in triglycerides (MD -15.34, 95%CI -20.37, -10.31, I² =0%, p<0.001, n=5) (**Figure 5B**) and fasting glucose (MD -9.08, 95%CI -15.29, -2.88, I² = 95%, p=0.004, n=3) (**Figure 6A**) among dyslipidemic volunteers.

**Figure 7:**
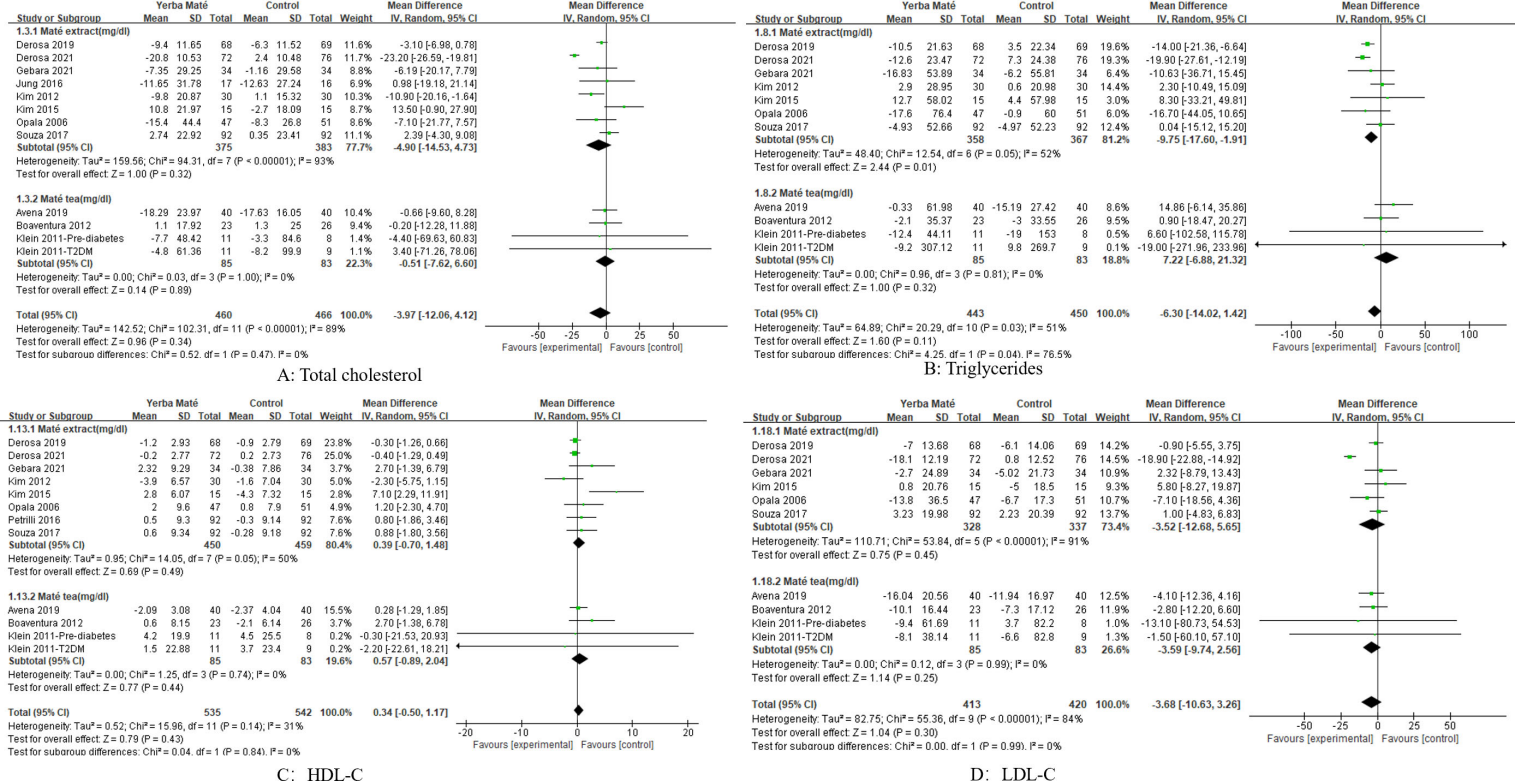
Forest plot for subgroup analysis of intervention methods on lipid levels. **(A)** Total cholesterol; **(B)** Triglycerides; **(C)** HDL-C (high-density lipoprotein cholesterol); **(D)** LDL-C (low-density lipoprotein cholesterol).

We conducted a subgroup analysis of our intervention methods for Yerba Maté (**Figure 7**), including Yerba Maté extract and Yerba Maté. In this subgroup analysis, the intervention with Yerba Maté extract significantly reduced triglycerides(MD -9.75, 95%CI -17.60, -1.91, I² =52%, p=0.01, n=7) (**Figure 7B**). Most intervention methods included in the study were Yerba Maté extracts, with no more than one study involving Yerba Maté tea. Therefore, no subgroup analysis of intervention methods based on fasting glucose, waist circumference, and BMI was conducted.

**Figure 8:**
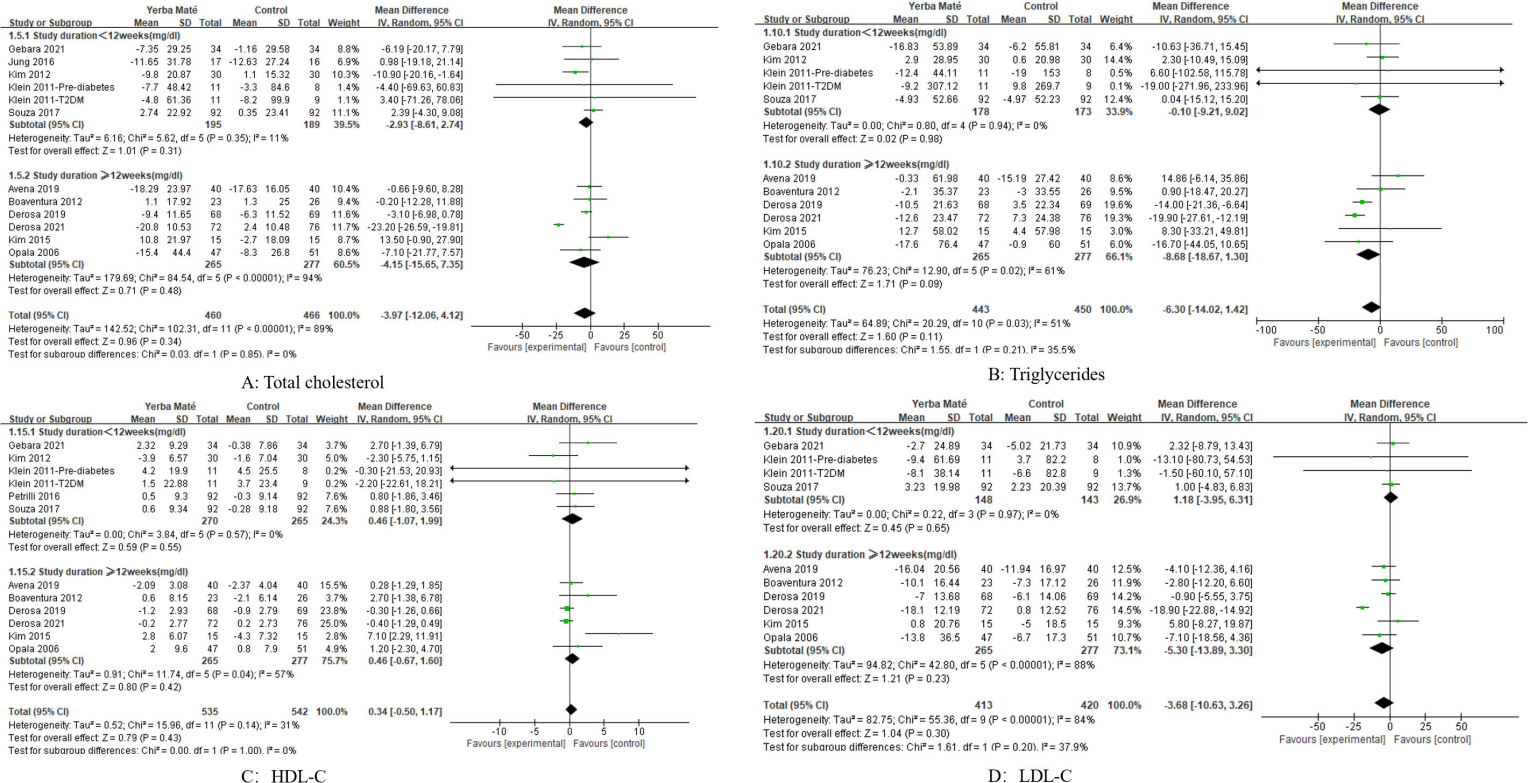
Forest plot for subgroup analysis of intervention duration on lipid levels. **(A)** Total cholesterol; **(B)** Triglycerides; **(C)** HDL-C (high-density lipoprotein cholesterol); **(D)** LDL-C (low-density lipoprotein cholesterol).

**Figure 9:**
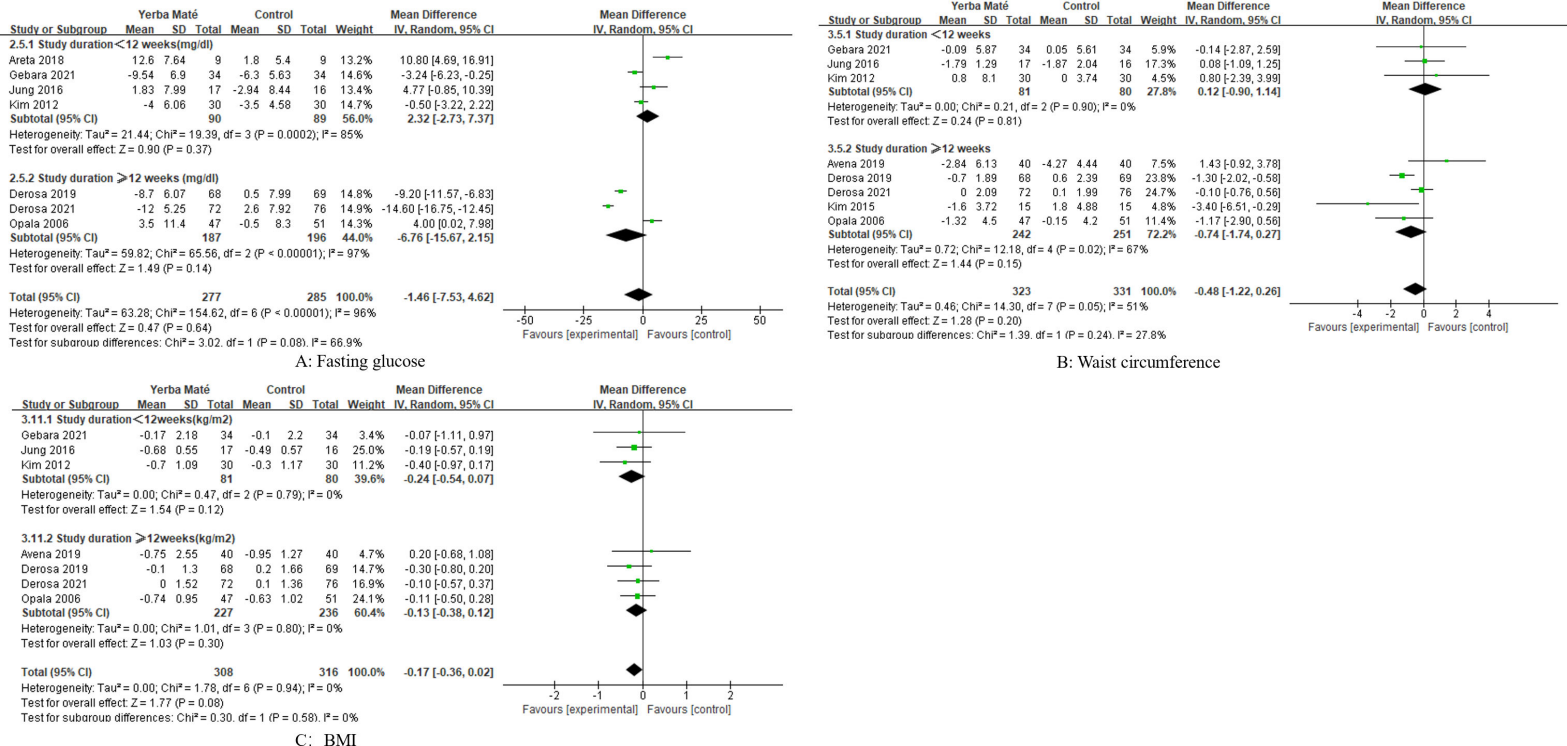
Forest plot for subgroup analysis of intervention duration on fasting glucose, waist circumference and BMI. **(A)** Fasting glucose, **(B)** Waist circumference, **(C)** BMI (body mass index).

We conducted a subgroup analysis of the intervention duration for Yerba Maté, including periods of less than 12 weeks and 12 weeks or longer. However, no significant differences were found (**Figures 8**, **9**).

### Adverse events

The safety profile of Yerba Maté was reported in four studies (12, 18, 21, 49), with adverse effects summarized in **Table 4**. Yerba Maté tea consumption has been associated with adverse effects in certain individuals, including oral or gastric mucosal irritation, insomnia, nausea, acid reflux, tachycardia, angina pectoris, headache, and abdominal discomfort.

**Table 4:** Adverse effects of Yerba Maté.

First author, year [reference]	Number of patients	Number of adverse events	Adverse effects
MORAIS, 2009 (12)	118	4	irritation of the oral or stomach mucosa, insomnia, or nausea
Klein, 2011 (17)	85	8	insomnia, heartburn, and tachycardia
Kim, 2015 (21)	30	1	adverse side effects
Opala, 2006 (49)	98	8	gastrointestinal discomfort

### Sensitivity analyses

**Figure 10:**
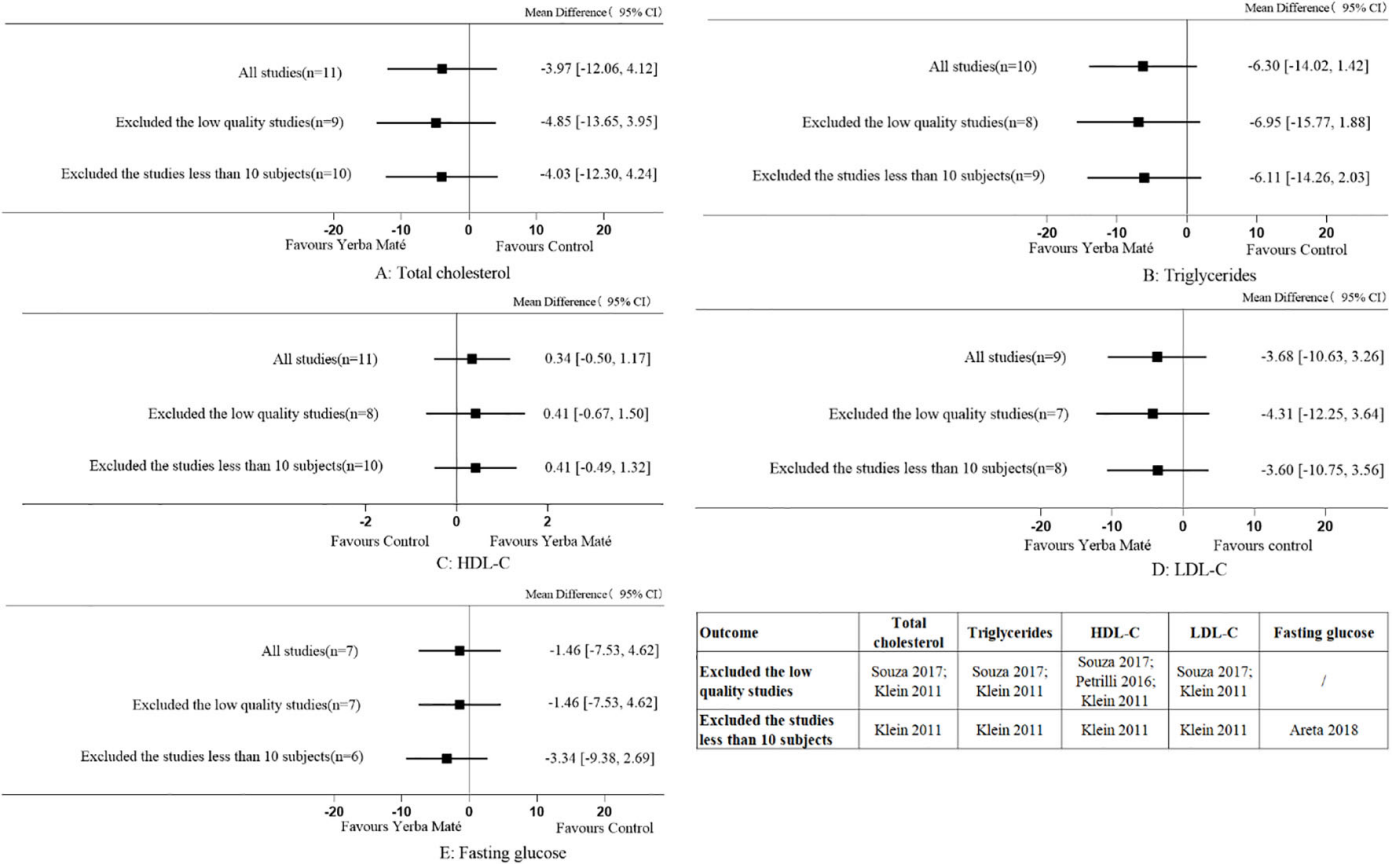
Sensitivity analyses for Yerba Maté consumption on lipid levels and fasting glucose. **(A)** Total cholesterol; **(B)** Triglycerides; **(C)** HDL-C (high-density lipoprotein cholesterol); **(D)** LDL-C (low-density lipoprotein cholesterol), **(E)** Fasting glucose.

To evaluate the reliability of the results regarding Yerba Maté’s effects on lipid profiles and fasting blood glucose levels, sensitivity analyses were performed (**Figure 10**). Excluding low-quality studies and those with fewer than 10 participants did not alter the overall findings, confirming the robustness of the meta-analysis results. No sensitivity analysis was performed on fasting insulin, postprandial glucose, HbA1c, HOMA index, waist circumference and BMI, since the studies did not include low-quality research or studies with fewer than 10 participants.

## Discussion

### Principal findings

Our systematic review and meta-analysis demonstrate that Yerba Maté consumption may improve glycemic outcomes, including decreased postprandial glucose levels, HbA1c levels, and the HOMA index. However, no significant effects were observed on lipid profiles (triglycerides, total cholesterol, HDL-C, LDL-C), waist circumference, or BMI. These findings suggest that Yerba Maté may exert glycemic control benefits independent of lipid metabolism modulation.

It is important to highlight that the two studies indicating Yerba Maté’s beneficial effects on blood glucose were conducted by the same research team (44, 45), which may introduce reporting bias and warrants cautious interpretation. In these two studies (44, 45), the subjects were individuals with impaired fasting glucose and impaired glucose tolerance. While other studies (41, 46–49) included diverse populations such as healthy volunteers, athletes, and overweight individuals with normal or well-regulated blood glucose, who may respond less noticeably to interventions, leading to non-significant results. In addition, these two studies employed a longer intervention period of three months, providing a more extended window to observe potential changes in blood glucose indicators, whereas some shorter-term studies may not have been as effective in capturing significant improvements (41, 46–48). However, it should be noted that the two studies were conducted by the same research team and used a standardized formulation produced by the same company. This might lead to reporting bias. Therefore, their positive results should be interpreted with caution.

To contextualize our findings, we compared the effect sizes for fasting glucose and triglycerides with those reported for cinnamon and fenugreek (52, 53). Yerba Maté and cinnamon achieve identical fasting-glucose reductions (MD -9.08), surpassing fenugreek (MD -0.84); for triglycerides cinnamon (54) retains the largest effect (WMD -29.59), while yerba maté provides a moderate but clinically relevant benefit (MD -20.37).

Some systematic reviews have yielded inconsistent results concerning the metabolic impacts of Yerba Maté supplementation. For example, Masson et al. (10) showed a significant reduction in LDL-C in non-diabetic populations, Clemente et al. (15) found HDL-C improvements in obese populations using 3g/d standardized extracts, but José et al. (55) observed no effects on glycemic parameters in type 2 diabetes patients given <1g/d extracts. Four factors might account for the disparity between our findings and prior meta-analyses. First, there are population-specific differences in metabolic pathways. José et al. (55) focused on chronic disease populations but excluded the acute metabolic responses in our athlete subgroup. While Clemente et al. (15) reported HDL-C improvement in homogeneous obese cohorts, our study included HIV patients and athletes whose lipid metabolism may be affected by antiretroviral therapy (50) or exercise-induced adaptations (41). Second, dose-response heterogeneity is crucial. Unlike Luís et al. (21), who focused on 3g/d standardized extracts, our analysis included extreme dosing like 50g/d tea in Avena 2019 (42). Third, there were marked differences in outcome measurement timelines. The HbA1c reduction in our study mainly came from ≥84-day interventions (44, 45), whereas the negative waist circumference findings included short-term trials like the 5-day cycling protocol in Areta 2018 (41), which contrasts with Luís et al.’s (21) 90-day focused analysis. Fourth, heterogeneity in study design caused bias. Our review strictly included only parallel RCTs and crossover RCTs to maximize internal validity. In contrast, Masson et al. (14) combined RCTs and observational studies (e.g., cohort and case-control studies), which might have allowed lifestyle confounders such as diet and exercise to interfere with the independent link between Yerba Maté intervention and metabolic outcomes.

### Subgroup analysis

Subgroup analysis of the intervention population revealed significant differences in triglycerides and fasting glucose levels among dyslipidemic volunteers. Subgroup analysis of the intervention methods revealed Yerba Maté extracts were significantly differences in triglycerides. Dyslipidemic volunteers and Yerba Maté extracts as possible sources of heterogeneity. Future studies may explore larger-scale interventions with Yerba Maté extracts among dyslipidemic volunteers. However, subgroup analysis conducted in subgroups according to different intervention duration showed no significant differences in total cholesterol, triglycerides, HDL-C, LDL-C, fasting glucose, waist circumference and BMI between Yerba Maté and control groups.

### Mechanism basis

Our systematic review and meta-analysis suggest that Yerba Maté consumption may be associated with improvements in glycemic control, as indicated by reductions in postprandial glucose, HbA1c levels, and HOMA index. However, no significant effects were observed on fasting glucose or insulin levels. These findings hint at potential mechanisms that might underlie the observed effects, such as enhanced insulin sensitivity and postprandial glucose regulation, though the exact mechanisms remain to be fully elucidated. Existing literature proposes that Yerba Maté polyphenols could potentially modulate hepatic insulin signaling by suppressing TNF-α (56), and may interact with the PI3K-AKT pathway, involving elements like Akt2 and Irs1, possibly contributing to improved peripheral insulin resistance (57). Regarding postprandial glucose control, it has been hypothesized that bioactive compounds in Yerba Maté might inhibit intestinal SGLT1-mediated glucose absorption (58). Additionally, its anti-glycation properties, which seem to surpass those of green tea (59), could potentially protect β-cells by inhibiting AGE formation during the secondary glycation phase. While these mechanistic insights offer plausible explanations for the observed effects, they are largely derived from preclinical studies and *in vitro* evidence. As such, they should be interpreted with caution and require further validation through well-designed clinical trials before Yerba Maté can be positioned as an adjuvant therapy for diabetes management.

The present study found no significant effects of Yerba Maté consumption on waist circumference or body mass index (BMI), suggesting limited efficacy in directly reducing abdominal adiposity or overall body weight. However, preclinical evidence indicates its potential to modulate energy metabolism through three interconnected mechanisms: (1) Caffeine-mediated activation of the sympathetic nervous system enhances lipolytic enzyme activity, though clinical relevance requires exceeding dose-dependent thresholds (>3 g/day of standardized extracts or >20 g/day of traditional infusion) to achieve thermogenic effects (60–62); (2) While acute interventions (e.g., 5-day trials) demonstrate efficacy in delaying gastric emptying and enhancing satiety via gastrointestinal modulation, these effects fail to translate into sustained body composition improvements in longitudinal studies, likely due to compensatory metabolic adaptations (28, 63); (3) Although Yerba Maté supplementation reduces chronic inflammation markers such as C-reactive protein (CRP) – a known correlate of visceral adiposity – its anti-inflammatory properties exhibit paradoxical dissociation from abdominal fat redistribution, necessitating extended clinical trials to validate causal relationships (55, 62).

### Potential adverse effects

Although Yerba Maté (Ilex paraguariensis) is recognized for its numerous health benefits, its potential adverse effects and carcinogenic risks warrant attention. Studies have indicated that long-term, high-volume consumption of Yerba Maté may increase the risk of certain cancers, including oral, esophageal, laryngeal, and lung cancers (64–68). Notably, the risk of carcinogenesis is significantly elevated when Yerba Maté is consumed at temperatures exceeding 65 °C, which may be attributed to thermal injury to esophageal cells caused by hot beverages (65–68). Additionally, Yerba Maté contains various chemical constituents, such as tannins, nitrosamines, and polycyclic aromatic hydrocarbons, which may possess carcinogenic potential (68–72). However, other research has suggested that the polyphenolic compounds in Yerba Maté may exert protective effects against certain cancers (70–72). Despite these findings, the current evidence is inconsistent, with some studies indicating that the association between hot Yerba Maté consumption and increased cancer risk may be influenced by other factors, such as smoking and alcohol consumption (64, 73). The randomized controlled trials included in this systematic review all had intervention periods of no more than three months. Future research necessitates long-term follow-up studies to further investigate the safety profile of Yerba Maté, particularly its health impacts under different consumption temperatures and dosages.

### Methodological quality of included trials

The methodological quality assessment revealed notable variations across study designs. Among the nine parallel RCTs, five studies (21, 44, 45, 48, 49) demonstrated low risk of bias across all domains, while the remaining four exhibited some concerns or high risk in specific domains (particularly randomization process and deviations from intended interventions). The crossover RCTs showed more substantial methodological limitations, with two studies (50, 51) classified as high risk and two (41, 46) having some concerns across multiple domains.

### Sources of heterogeneity

Significant heterogeneity persisted in this study’s meta-analysis, despite conducting subgroup and sensitivity analyses. Several factors likely contributed to this heterogeneity. First, the studies encompassed a wide range of participant characteristics, including age, gender, and health status, from healthy individuals to those with conditions like obesity, prediabetes, metabolic syndrome, and HIV infection. Such diversity in metabolic status and disease burden can lead to varied responses to Yerba Maté interventions. Second, differences in the form of intake of Yerba Maté and study design, such as differences in the mode of use (tea infusions, extract or capsules), whole and part amounts used, dosage, and duration of intervention, could act as confounders responsible for differences between effects in the short term and the long term. Third, studies were marked by strong differences in baseline characteristics. Some studies may have included subjects with high baseline triglyceride levels. In contrast, others have lower baseline levels that could have resulted in different effects of Yerba Maté being observed. Finally, heterogeneity may be attributed to study design and methodology differences, e.g., randomization, blinding and data collection. These issues can be addressed by future research that standardizes intervention and measurement protocols, broadens the study population, controls all confounding variables, and strengthens the study’s design. Since this approach reduces heterogeneity, it will aid in increasing the reliability and the generalizability of findings.

### Strengths and limitations

The present study has several strengths. Its exhaustive search strategy allows all relevant literature to be included. By minimizing the risk of publication bias, this approach guarantees that as many studies as possible are represented and thus makes the findings more robust. Moreover, the studies are assessed with rigorous evaluation methods, including the RoB 2 tool, for the risk of bias so that each study is thoroughly scrutinized. The GRADE approach adds to the analysis by practicing a systematic and transparent approach to evaluating the overall quality of evidence and, thus, the level of confidence in the results.

This review is subject to certain limitations. Given that research findings from South America may be published in languages such as Spanish, we ultimately decided to include studies without language restrictions. This is not consistent with our PROSPERO record. The major issue is a big heterogeneity in clinical and procedural aspects of the Yerba Maté consumption in the studies examined, including differences in the details of their participants, their Yerba Maté consumption regimens, and the different follow-up durations. Further, heterogeneity extends to assessing such outcomes as total cholesterol, LDL-C, fasting blood glucose, postprandial blood glucose, HOMA index and BMI, which may influence the comparison and the generalizability of the results. In addition, the study of outcomes, including postprandial blood glucose, HOMA index, and HbA1c, is limited to only two of the included studies, which may result in too few subjects to allow sound conclusions. Furthermore, three studies had a follow-up period under 20 days, which may raise a question of the length of follow-up was insufficient for the Yerba Maté consumption. Future research should strive to develop protocols and outcome measures that are standardized in order to facilitate comparison and reliability of results.

### Implications for future research

Although consumption of Yerba Maté has shown health benefits, future research should go further in studying the long-term effects and risks of this consumption. Future research investigations on Yerba Maté and glycemic control should focus on extending the intervention duration, employing Yerba Maté extracts, and enrolling subjects with metabolic disorders. As far as it is carcinogenic, this matter is still inconclusive because some studies on Yerba Maté show that carcinogenic substances (Polycyclic aromatic hydrocarbons, or PAHs, and tannins) come not from Yerba Maté itself but from the contamination during processing. Yet more studies have come to the conclusion that perhaps it is not the tea that poses a problem but the high temperature with which it is traditionally consumed. Moreover, smoking and alcohol drinking may contribute to the observed cancer risk. Given the present study’s limitations, large-scale prospective cohort studies are required to clarify whether Yerba Maté carries a carcinogenic risk and to differentiate temperature, PAH, and other variables.

Furthermore, the role of Yerba Maté in blood pressure and other metabolic syndrome indices (uric acid) has been widely ignored. The current research focus mainly involves short-term interventions with limited systematic evaluation of long-term effects. RCTs that are well designed are necessary to validate the effects of Yerba Maté on the metabolic indicators and elucidate the physiological mechanisms. Evaluation of the role of Yerba Maté in preventing and treating chronic diseases has not been possible because of the lack of long-term intervention studies. Studies of the incidence of severe cardiovascular events in prospective cohort studies with extended follow-up could supply important information about its long-term health effects.

Then, the possible effects of Yerba Maté on several different populations, e.g., the older adults, the ones who are athletes, and the diabetic, are variant due to the differences in each person. Its mechanisms of action and effects in specific populations merit future research, focusing on the feasibility of a personalized intervention. In doing this, we will augment our entity’s understanding of the health implications of Yerba Maté.

## Conclusion

In summary, our systematic review and meta-analysis suggest that Yerba Maté consumption may significantly reduce postprandial glucose, HbA1c, and HOMA index. However, it did not show significant impacts on total cholesterol, triglycerides, HDL-C, LDL-C, fasting glucose, fasting insulin, waist circumference, or BMI, and further research is needed to confirm these preliminary findings. Although some adverse events were reported, the overall findings suggest that Yerba Maté may exert glycemic control benefits independent of lipid metabolism modulation. Future research should address the identified limitations and knowledge gaps through large-scale, rigorously designed RCTs and longitudinal cohort studies.

## Data Availability

The original contributions presented in the study are included in the article/**Supplementary Material**. Further inquiries can be directed to the corresponding author.
