# Supplementary material for: Yerba Maté and its impact on glycemic control and metabolic health: a systematic review and meta-analysis
Source: Front Endocrinol (Lausanne). 2025 Oct 30;16:1641592. doi: 10.3389/fendo.2025.1641592 (PMC12611702; doi:10.3389/fendo.2025.1641592)

**Supplementary Figure 1.** Risk of bias assessment in parallel RCT trials.


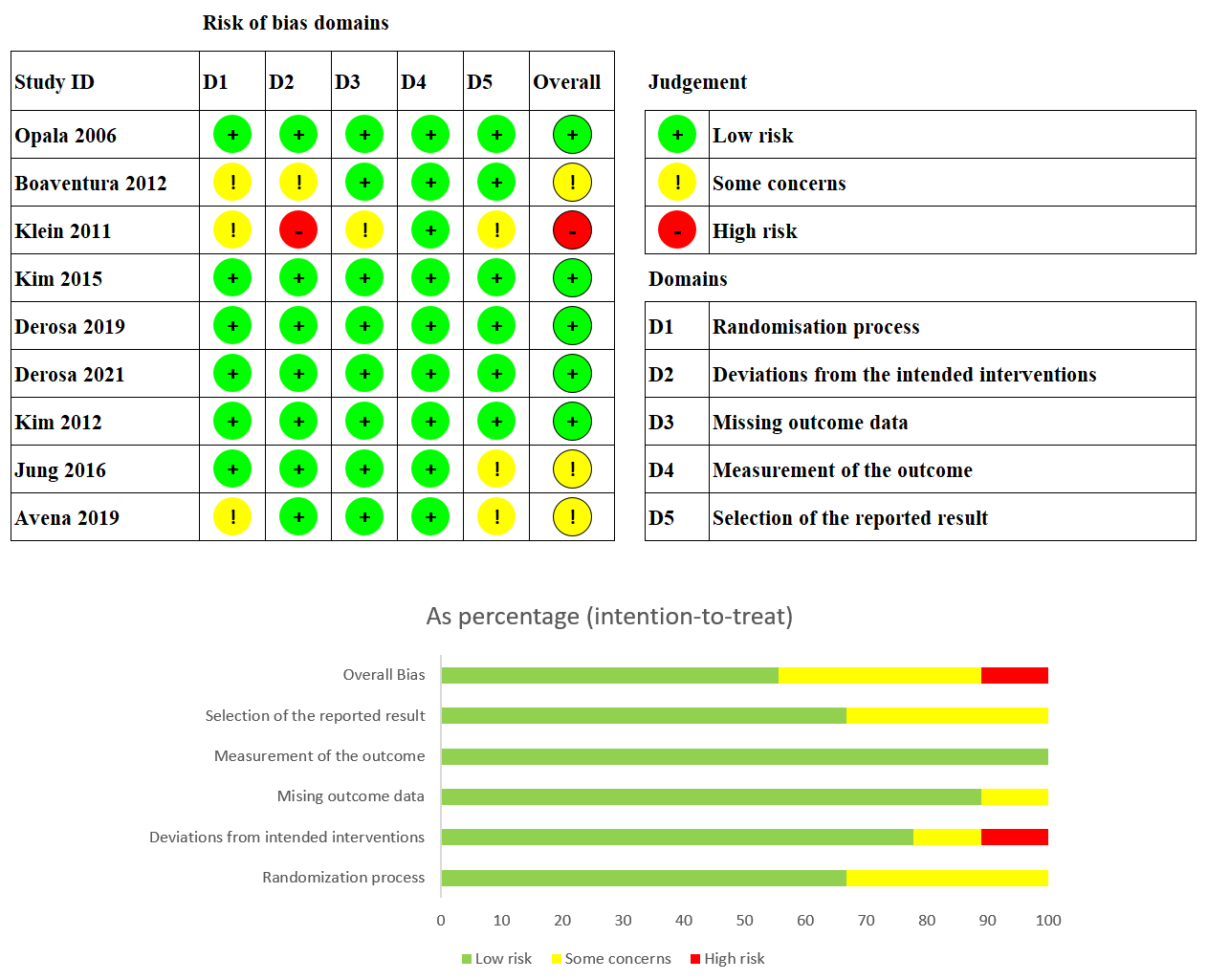


**Supplementary Figure 2.** Risk of bias assessment in crossover trials.


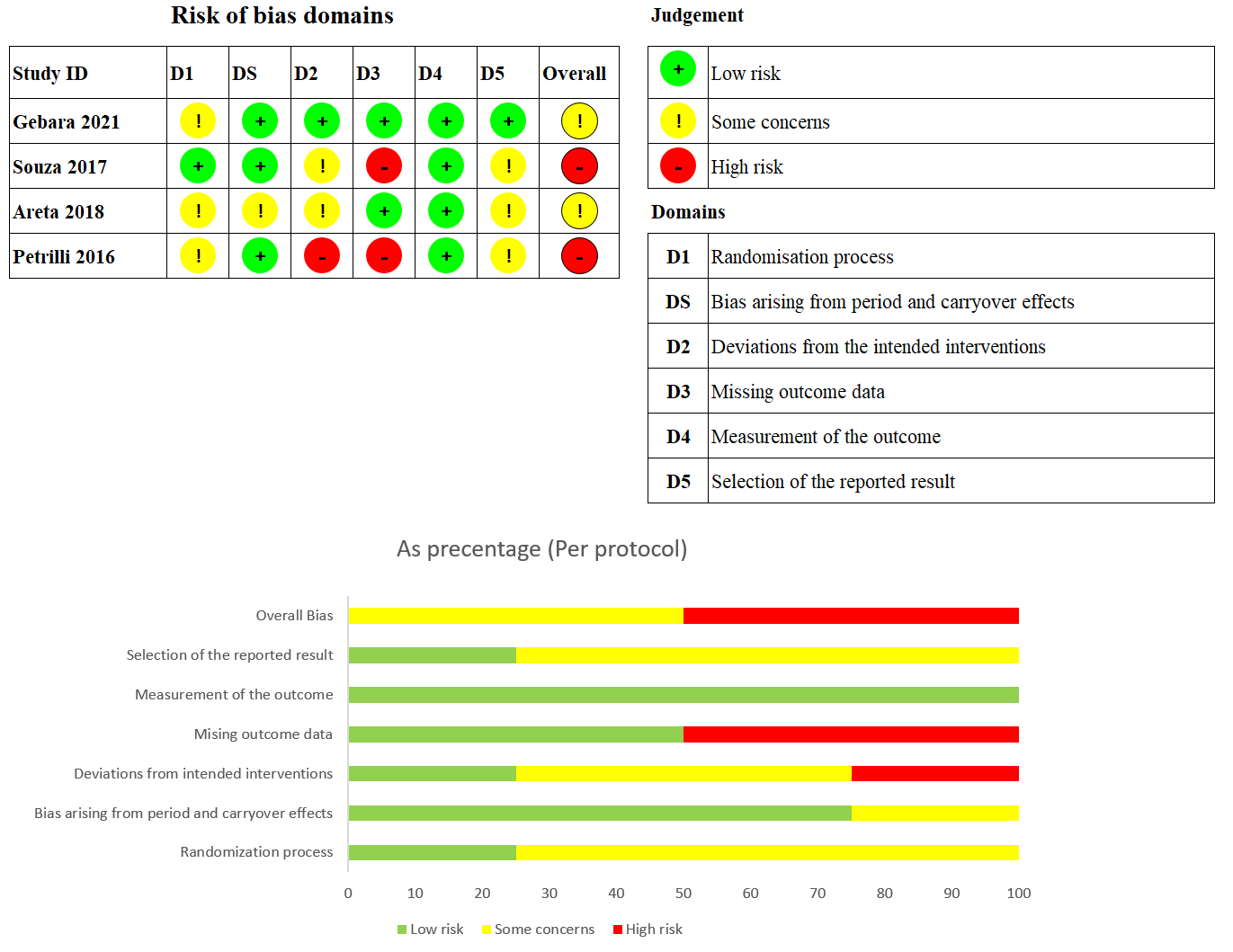

Supplement: Supplementary file 1 [file DataSheet1.docx]
