# Supplementary material for: Yerba Maté and its impact on glycemic control and metabolic health: a systematic review and meta-analysis
Source: Front Endocrinol (Lausanne). 2025 Oct 30;16:1641592. doi: 10.3389/fendo.2025.1641592 (PMC12611702; doi:10.3389/fendo.2025.1641592)
Supplement: Supplementary file 2 [file DataSheet2.docx]

**Supplementary Table 1**

The Detailed Search Strategy Via OVID SP on January 14, 2025

| EBM Reviews-Cochrane Central Register of Controlled Trials (December 2024), EMBASE (1974 to 2025 January 10), Ovid MEDLINE(R) and Epub Ahead of Print, In-Process, In-Data-Review & Other Non-Indexed Citations, Daily and Versions(R) (1946 to January 10, 2025) | | |
| --- | --- | --- |
| 1 | exp ilex paraguariensis/ | 1214 |
| 2 | ("mate" or "chimarrao" or "ilex paraguariensis" or "terere" or "yerba-mate").ab. | 29054 |
| 3 | randomized controlled trial.pt. | 629887 |
| 4 | controlled clinical trial.pt. | 95676 |
| 5 | randomized.ab. | 2411298 |
| 6 | placebo.ab. | 994745 |
| 7 | drug therapy.fs. | 7686402 |
| 8 | random*.ab. | 4804596 |
| 9 | trial.ab. | 2456119 |
| 10 | groups.ab. | 7313998 |
| 11 | 3 or 4 or 5 or 6 or 7 or 8 or 9 or 10 | 17520821 |
| 12 | exp animals/ not humans.sh. | 38176331 |
| 13 | 11 not 12 | 7893080 |
| 14 | 1 or 2 | 29198 |
| 15 | 13 and 14 | 1451 |

**Supplementary Table 2 Reasons for exclusions**

| 1. | Alkhatib,2014^26^ | Intervention | Reported outcome data of FAO, CHO, EE, RER and VO_2 Peak_ |
| --- | --- | --- | --- |
| 2. | Alkhatib,2015^27^ | Intervention | Reported outcome data of FAO |
| 3. | Alkhatib,2017^28^ | Intervention | Reported outcome data of FAO, CHO, EE, HR, VAS and POMS |
| 4. | Andersen,2001^29^ | Intervention | reported data before and after the intervention |
| 5. | Balsan,2019^30^ | Intervention | Not reported lipid parameters after the intervention |
| 6. | Filipini,2024^31^ | Intervention | Reported outcome data of FMD, BP, HR, HRV |
| 7. | Harrold,2013^32^ | Intervention | Reported outcome data of VAS |
| 8. | Morais,2009^12^ | Design | Not randomized |
| 9. | Martinet,1999^33^ | Intervention | Reported outcome data of EE and RQ |
| 10. | Maufrais,2018^34^ | Intervention | Reported outcome data of EE, HR, skin blood flow and hand temperature |
| 11. | Min,2005^35^ | Intervention | Reported outcome data of electrocardiographic (ECG) |
| 12. | Pagliosa,2021^36^ | Intervention | Reported outcome data of serum iron |
| 13. | Panza,2016 ^37^ | Intervention | Reported outcome data of total phenolics, GSH, GSSG, GSH:GSSG ratio and lipid hydroperoxides (LOOH) |
| 14. | Panza,2018^38^ | Intervention | Reported outcome data of CD16+/CD14- cells, interleukin (IL)-1β (IL-1β), tumour necrosis factor-alpha (TNF-α), IL-6, total phenols, and reduced and oxidised glutathione (GSH and GSSG, respectively) |
| 15 | Schubert,2013^39^ | Intervention | Reported outcome data of VO2max |
| 16 | Yu,2015^40^ | Intervention | Reported outcome data of triglycerides, total cholesterol, HDL-C, LDL-C in the form of pictures, without specific data |

FAO: fatty acid oxidation, CHO: carbohydrate oxidation, EE: energy expenditure, RER: espiratory exchange ratio, VO_2 Peak_ :peak oxygen uptake; COP: the cross-over point, HR: heart rate, VAS: Visual Analogue Scale, POMS: Profile of Mood States, FMD : flow-mediated dilation , BP : blood pressure, HRV: heart rate variability, RQ : respiratory quotient, HDL-C: high-density lipoprotein cholesterol, LDL-C: low-density lipoprotein cholesterol, HbA1c: glycated hemoglobin, HOMA index: homeostatic model assessment index, BMI: body mass index, AEs: adverse events
