# Supplementary material for: Yerba Maté and its impact on glycemic control and metabolic health: a systematic review and meta-analysis
Source: Front Endocrinol (Lausanne). 2025 Oct 30;16:1641592. doi: 10.3389/fendo.2025.1641592 (PMC12611702; doi:10.3389/fendo.2025.1641592)

**Appendix 1 Protocol Amendments**

The protocol posted on PROSPERO included dyslipidaemia and an English-only language limit. After retrieval we narrowed the review to glycaemic outcomes (dyslipidaemia data extracted only for descriptive safety) and removed the language restriction to capture South-American literature.

**Appendix 2 Cross-over vs parallel subgroup analyses**

In addition, sources of heterogeneity will be explored by Cross-over vs parallel subgroup analyses and meta-regression where ≥5 studies are available. Because the comparison was under-powered (only 4 cross-over trial) and added no meaningful information, we omitted it from the main tables but have now included it in Appendix 2 for transparency. Overall lipid effect did not differ between designs: total cholesterol (parallel: MD -4.56, 95% CI -13.80, 4.68; cross-over: MD 0.39, 95% CI -6.73, 7.50), .triglycerides (parallel: MD -6.62, 95% CI -15.67, 2.43; cross-over: MD -2.65, 95% CI -15.76, 10.45), HDL-C (parallel: MD 0.18, 95% CI -0.81, 1.18; cross-over: MD 1.17, 95% CI -0.55, 2.88) and LDL-C (parallel: MD -5.37, 95% CI -13.62, 2.89; cross-over: MD 1.29, 95% CI -3.88, 6.45). Overall blood glucose metabolism effect did not differ between designs fasting glucose (parallel: MD -3.32, 95% CI -10.57, 3.93; cross-over: MD 3.52, 95% CI -10.23, 17.27). Overall weight management effect did not differ between designs waist circumference (parallel: MD -0.50, 95% CI -1.30, 0.30; cross-over: MD -0.14, 95% CI -2.87, 2.59) and BMI (parallel: MD -0.18, 95% CI -0.37, 0.02; cross-over: MD -0.07, 95% CI -1.11, 0.97).


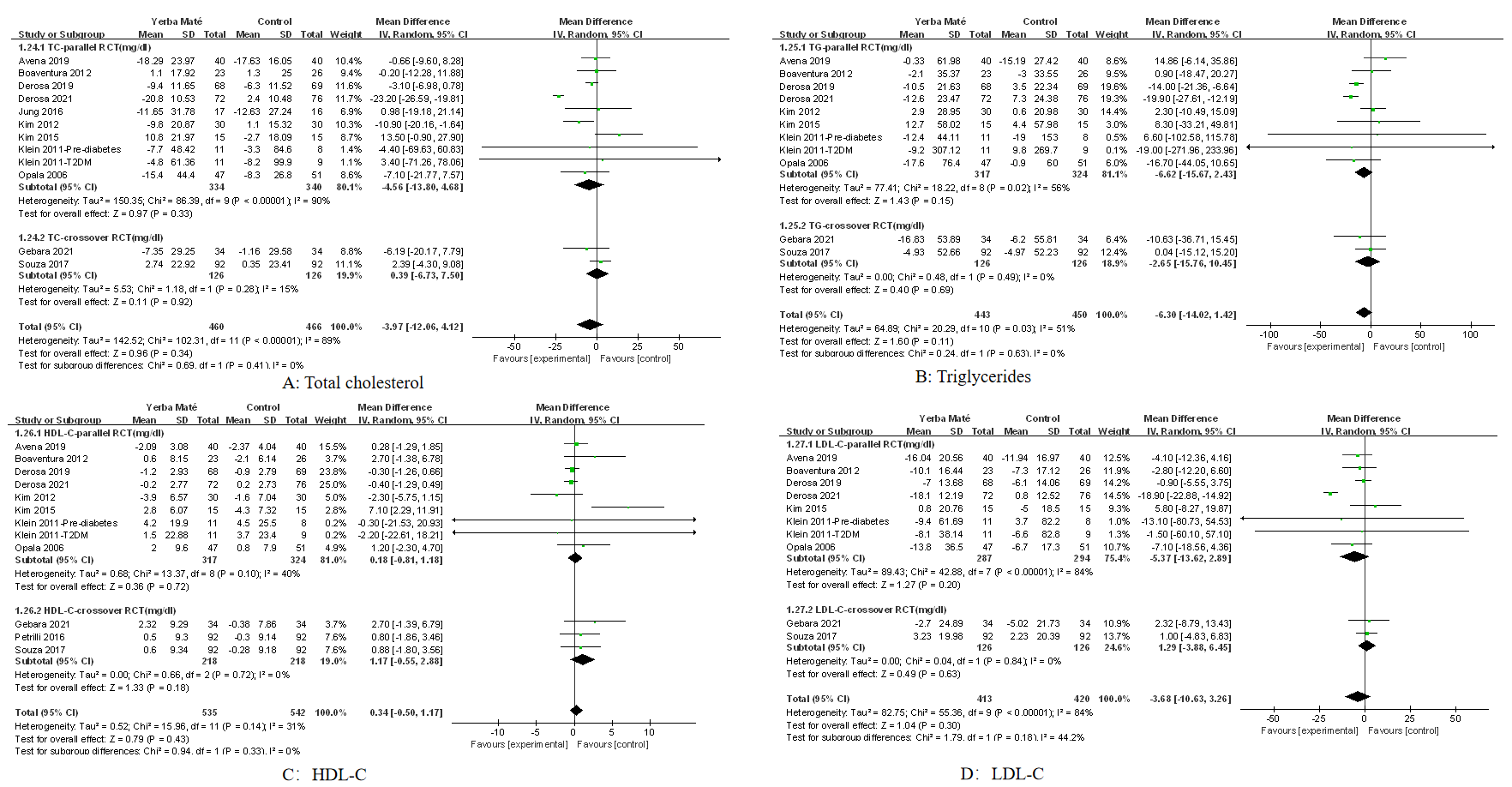


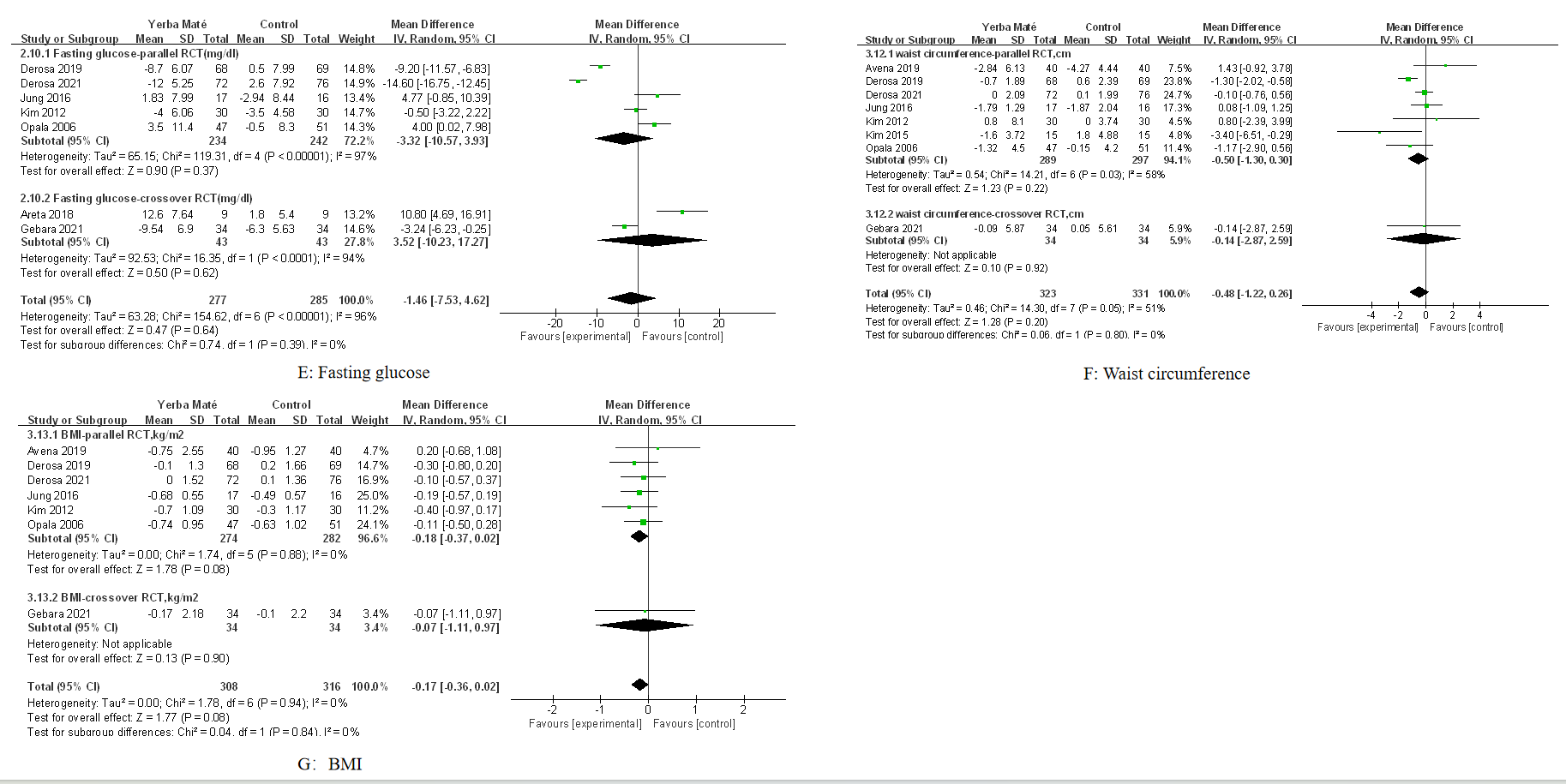

Supplement: Supplementary file 3 [file DataSheet3.doc]
